# *CYP1B1* mutations in Spanish patients with primary congenital glaucoma: phenotypic and functional variability

**Published:** 2009-02-23

**Authors:** Ezequiel Campos-Mollo, María-Pilar López-Garrido, Cristina Blanco-Marchite, Julián Garcia-Feijoo, Jesús Peralta, José Belmonte-Martínez, Carmen Ayuso, Julio Escribano

**Affiliations:** 1 Servicio de Oftalmología, Hospital General Universitario de Alicante, Alicante, Spain; 2 Área de Genética, Facultad de Medicina/Centro Regional de Investigaciones Biomédicas (CRIB), Universidad de Castilla-La Mancha, Albacete, Spain; 3 Cooperative Research Network on Age-Related Ocular Pathology, Visual and Life Quality, Instituto de Salud Carlos III, Madrid, Spain; 4 Servicio de Oftalmología, Complejo Hospitalario Universitario de Albacete (Hospital Perpetuo Socorro), Albacete, Spain; 5 Servicio de Oftalmología, Hospital San Carlos, Madrid, Spain; 6 Servicio de Oftalmología Infantil, Hospital La Paz, Madrid, Spain; 7 Servicio de Genética, Fundación Jiménez Díaz, CIBERER, Madrid, Spain

## Abstract

**Purpose:**

To analyze the contributions of cytochrome P4501B1 (*CYP1B1*) mutations to primary congenital glaucoma (PCG) in Spanish patients.

**Methods:**

We analyzed, by polymerase chain reaction (PCR) DNA sequencing, the presence of promoter (−1 to −867) and exon *CYP1B1* mutations in 38 unrelated Spanish probands affected by PCG. Functional analysis of nine identified mutations was performed measuring ethoxyresorufin O-deethylation activity and *CYP1B1* stability in transiently transfected human embryonic kidney 293T (HEK-293-T) cells.

**Results:**

We found a total of 16 different mutations in 13 (34.2%) index cases. The identified mutations included nine missense and three nonsense nucleotide changes, three small deletions, and a short duplication. Eleven probands were compound heterozygotes and two were heterozygotes. Six of the identified mutations were novel (A106D, E173X, F261L, E262X, W341X, and P513_K514del). Mutations T404fsX30 and R355fsX69 were the most prevalent among index cases and were detected in six (23.0%) and three (11.5%) patients, respectively. Functional analysis showed that the three nonsense mutants assayed (E173X, E262X, and W341X) and F261L were null alleles. Of the remaining mutants, four (P52L, G61E, Y81N, and E229K) showed catalytic activities ranging from 20% to 40% of wild-type CYP1B1 and high protein instability. Mutation P400S showed normal catalytic activity and moderate instability. These five mutants were classified as hypomorphic alleles. Patients carrying two null alleles showed severe phenotypes featured by very early PCG onset usually at birth or in the first month of life (0.6±0.9 months). Incomplete penetrance was detected in patients carrying hypomorphic alleles.

**Conclusions:**

Our data indicate that approximately one-third of Spanish patients with PCG carry loss-of-function *CYP1B1* and show that null alleles are associated with the most severe phenotypes. Hypomorphic alleles may contribute to some cases of incomplete penetrance.

## Introduction

Primary congenital glaucoma (PCG; OMIM 231300) is usually transmitted as an autosomal-recessive trait with incomplete penetrance [1,2]. The disease is featured by developmental defects of the trabecular meshwork (TM) and the anterior chamber angle of the eye, which leads to aqueous outflow obstruction, elevated intraocular pressure (IOP), and optic nerve damage [3]. PCG is the most common childhood glaucoma, which is observed in the neonatal or infantile period, and is an important cause of visual loss in children. Parental consanguinity is frequently reported, mainly in Arab populations [4-6]. Its incidence has been reported to range from 1:1250 and 1:2500 births in inbred Slovakian Gypsy [7] and Saudi Arabian populations [2], respectively, to 1:5000 and 1:10000 births in Western countries [8].

Three PCG loci have been mapped, *GLC3A* (2p21) [9], *GLC3B* (1p36) [10], and *GLC3C* (14q24.3) [11]. To date, only the gene linked to the GLC3A locus, cytochrome P4501B1 (*CYP1B1*; OMIM 601771), has been identified [12]. Nonetheless, mutations in myocilin (*MYOC*;**OMIM 601652) [13,14] and forkhead box C1 (*FOXC1*; OMIM 601090) [15] have also been reported in some PCG cases. Although the mutations in *CYP1B1* are the main known genetic cause of this type of glaucoma [16] in different worldwide populations [4,6,17-22], the role of this gene in Spanish patients has not yet been investigated. Ninety-two *CYP1B1* mutations have now been implicated in the pathogenesis of PCG including missense, nonsense, frameshift mutations, and small insertions and deletions (the Human Gene Mutation Database professional release 8.1). Most missense mutations map to highly conserved functional regions of the protein. *CYP1B1* mutations are also present in certain families where PCG and primary open-angle glaucoma (POAG) coexist [5,21,23], in non-Mendelian POAG cases [24-26], and in monogenic anterior segment dysgenesis like Peters’ anomaly [27,28] and Rieger’s anomaly [29]. This gene has also been reported to act as a modifier gene in juvenile open-angle glaucoma [30]. The wide range of glaucoma phenotypes associated with *CYP1B1* mutations suggests that it plays a key role in the physiology and development of the eye [31].

*CYP1B1* encodes a member of the cytochrome P450 superfamily, subfamily I, and is composed of three exons with the translated region beginning at the 5′ end of the second exon. The CYP1B1 protein is a membrane-bound monomeric mixed function monooxygenase. This cytochrome has been proposed to participate in iridocorneal angle development [32], thus alteration of CYP1B1 catalytic activity could impair the morphogenesis of the outflow angle, leading to IOP elevation and glaucoma. In fact, partial loss-of-function, reduced stability, and diminished localization in the mitochondria have been described for *CYP1B1* mutations by a few previous functional reports [6,33-35].

We have herein investigated for the first time the role of *CYP1B1* mutations in a group of 38 unrelated Spanish families with PCG. We found 16 different loss-of-function *CYP1B1* mutations present in approximately one-third of the index cases. Our data show that complete loss of CYP1B1 catalytic activity is associated with the most severe phenotypes and indicate the influence of modifier genes and/or environmental factors in the phenotypic expression, particularly in subjects carrying hypomorphic *CYP1B1* alleles.

## Methods

### Subjects

A total of 39 patients (69 eyes) with PCG from 38 different families were recruited for genetic analysis. Thirty-eight patients were Spanish natives with Spanish family names, although the father of one of the index cases (PCG 41) was German. Patients with pediatric glaucoma, which was either related to other ocular disorders or associated with systemic abnormalities, were excluded. In addition, 70 unaffected patients’ relatives were studied. The study was approved by the Ethics Committees for Human Research of the hospitals involved (Hospital General Universitario de Alicante, Alicante, Spain, Hospital San Carlos, Madrid, Spain, Hospital La Paz, Madrid, Spain, and Fundación Jiménez Díaz, Madrid, Spain) and followed the tenets of the Declaration of Helsinki. Informed consents were obtained from all subjects included in the study. Clinical data were retrospectively reviewed. All subjects were clinically evaluated by glaucoma specialists. The ophthalmic examination included slit lamp biomicroscopy, gonioscopy, measurement of IOP and ophthalmoscopy. The clinical diagnosis included at least two of the following clinical features: increased corneal diameter (>12 mm) along with elevated IOP (>21 mmHg or >16 mmHg under general anesthesia) and/or Haab’s striae, corneal edema, and optic disc changes. The age at diagnosis ranged from 0 to 28.5 months, although in one case the diagnosis was delayed. Intraocular pressure was measured using the Perkins applanation tonometer generally under halothane anesthesia as soon as the child was sufficiently anesthetized to check intraocular pressure (during the first 10 min after induction and before tracheal intubation). Older and more cooperative children were assessed using a slit lamp for tonometry with a Goldman applanation tonometer. A control group composed of 325 patients from whom glaucoma was ruled out was used to screen for the presence of novel *CYP1B1* mutations identified in PCG patients. These subjects were recruited from among those who attended the clinic for conditions other than glaucoma including cataracts, floaters, refractive errors, and itchy eyes.

### Mutation analysis

Genomic DNA was extracted from the peripheral leukocytes of all the subjects studied using the QIAamp DNA Blood Mini Kit (Qiagen, Hilden, Germany) according to the manufacturer’s protocol. The translated (exons II and III), untranslated (exon I), and promoter (nucleotides −1 to −867) regions of  *CYP1B1* were amplified as previously described [26]. Terminator cycle sequencing was performed using the BigDye (v3.1) kit (Applied Biosystems, Foster City, CA), and the products of sequencing reactions were analyzed in an automated capillary DNA sequencer (ABI Prism 3130 genetic analyzer; Applied Biosystems). Mutations were confirmed by sequencing the cDNA strand and by independent sequencing in a second DNA sample. The haplotypes for the four SNPs (R48G, A119S, V432L, and N453S) were determined by segregation analysis.

### Site-directed mutagenesis

The cDNA encoding wild-type CYP1B1 was obtained from Invitrogen (cDNA clone ID 4662252; Carlsbad, CA) and was used as a template to produce different *CYP1B1* mutations found in PCG patients using the QuikChange site-directed mutagenesis kit (Stratagene, La Jolla, CA). The specific polymerase chain reaction (PCR) primers used for mutagenesis are presented in Table 1. The background haplotype of the wild-type cDNA clone for the four common coding SNPs (R48G, A119S, V432L, and N453S) was RAVN. Therefore, this was the background haplotype of all the cloned mutations. The cDNAs encoding the wild-type protein and the different mutant CYP1B1 forms were cloned into the Xba-BamHI sites of the pcDNA3.1-myc-His mammalian expression vector as previously described [36].

**Table 1:** Primer sequences used in this study.

**Primer set**	**Primer sequence (5′→3′)**
P52L	F: GGTCCGCGCCCCTGGGCCCGTTTGC
	R: GCAAACGGGCCCAGGGGCGCGGACC
G61E	F: GTGGCCACTGATCGAAAACGCGGCGGCG
	R: CGCCGCCGCGTTTTCGATCAGTGGCCAC
Y81N	F: CTGGCGCGGCGCAACGGCGACGTTTTC
	R: GAAAACGTCGCCGTTGCGCCGCGCCAG
E173X	F: CGGCTCTAGAATGGGCACCAGCCTCAGCCCG
	R: CGCAGGATCCGCGCTCAGCACGTGGCCCTCGAGG
E229K	F: CTGCTCAGCCACAACAAAGAGTTCGGGCGCAC
	R: GTGCGCCCGAACTCTTTGTTGTGGCTGAGCAG
F261L	F: CCGTTTTCCGCGAATTAGAGCAGCTCAACCGCA
	R: TGCGGTTGAGCTGCTCTAATTCGCGGAAAACGG
E262X	F: CGGCTCTAGAATGGGCACCAGCCTCAGCCCG
	R: CGCAGGATCCGCGAATTCGCGGAAAACGGTGCGC
P400S	F: TTGTGCCTGTCACTATTTCTCATGCCACCACTGCC
	R: GGCAGTGGTGGCATGAGAAATAGTGACAGGCACAA
R469W	F: TTTCAGTGGGCAAAAGGTGGTGCATTGGCGAAGAA
	R: TTCTTCGCCAATGCACCACCTTTTGCCCACTGAAA

In the "Primer sequence" column, "F" indicates forward primer and "R" indicated reverse primer.

### Cell transfections

The human embryonic kidney 293T (HEK-293T) cell line was obtained from the ATCC (American type Culture Collection, Manassas, VA), and it was maintained in Dulbecco’s modified Eagle’s medium (DMEM) supplemented with 10% fetal bovine serum (FBS) and antibiotics (penicillin and streptomycin; Invitrogen) at 37 °C in a fully humidified 5% CO_2_ atmosphere. Transient plasmid transfections were performed with 400 ng of total DNA using the Superfect Transfection Reagent (Quiagen, Valencia, CA) as previously described [37]. To assess transfection efficiency, the cDNA constructs encoding the different *CYP1B1 *mutants were cotransfected with 200 ng of a cDNA construct encoding the green fluorescent protein (GFP) from the jellyfish, *Aequorea victoria*. The GFP cDNA was cloned into the pcDNA3.1 vector. GFP was detected by western blot using an anti-GFP monoclonal antibody (Santa Cruz Biotechnology, Santa Cruz, CA).

### CYP1B1 enzymatic assay

The time course of CYP1B1 activity was analyzed by determining the ethoxyresorufin O-deethylation (EROD) activity in transfected HEK-293T cells. 7-Ethoxiresorufin substrate (400 nM) dissolved in PBS was added to cells in culture 13–15 h after transfection. After 2 h at 37 °C, the reaction product (resorufin) that secreted into the culture medium was measured in triplicate aliquots by a fluorometric assay [38] using a SpectraMax® GEMINI XS spectrofluorometer (Molecular Devices Corp, Sunnyvale, CA) at 530 nm (excitation) and 590 nm (emission). Endogenous CYP1B1 activity was estimated in control assays performed in HEK-293T cells transiently transfected with the non-recombinant pcDNA3.1 vector. The background activity was subtracted from all the enzymatic assays performed either with wild-type or mutant CYP1B1. Three independent assays for each mutant were performed.

### CYP1B1 stability assay

Protein stability was studied by western blot of transfected cells incubated with cycloheximide (10 µg/ml) for 0 h, 2 h, 5 h, and 8 h. Cycloheximide was added 13–15 h after transfection. After cycloheximide treatment, adhered cells were washed twice with 1 ml of DMEM followed by an addition of 200 µl of lysis buffer containing proteinase inhibitors. Collected cells were vortexed for 30 s at the maximum speed, incubated for 30 min on ice, and sonicated for 10 s (cycle 0.5 s). Aliquots of cell lysates were treated with loading buffer containing β-mercaptoethanol, boiled for 10 min, and fractionated by SDS–PAGE. Analytical 10% PAGE in the presence of SDS was performed using the Mini-PROTEAN III gel electrophoresis system (Bio-Rad, Hercules, CA). Gels were transferred onto Hybond ECL nitrocellulose membranes (Amersham, Uppsala, Sweden) for immunodetection. A commercial mouse monoclonal anti-myc antibody (Santa Cruz Biotechnology) was used as the primary antibody diluted at 1:500. A horseradish peroxidase-conjugated antibody against mouse IgG (Pierce Biotechnology, Rockford, IL) was diluted at 1:1000. Chemiluminescence detection was performed with Supersignal Dura Western Blot reagents (Pierce). The data from triplicate independent experiments were obtained. The amount of CYP1B1 protein was determined by densitometry, and the relative amounts at the different time points were expressed as a percentage of levels at time 0 h.

### Statistical analysis

Significance of the difference in age and IOP among patients was determined by the *t*-test. Data were statistically analyzed by using SigmaStat 2.0 software (SPSS Inc., Chicago, IL).

### Mutation nomenclature

Mutations were named based on the cDNA reference sequence U03688 [39]. The first nucleotide of the transcription initiation site is denoted as nucleotide *+*1 according to Tang and coworkers [40].

## Results

### Clinical phenotype of patients

To investigate the role of *CYP1B1* mutations in the development of PCG in Spanish patients, we retrospectively studied a total of 38 unrelated families affected by the disease. PCG segregated as an autosomal recessive trait in 37 families and showed a dominant or pseudo-dominant pattern in one family (PCG 35). Twenty-eight patients (72%) were male, and 11 patients (28%) were female. The disease was bilateral in 30 patients (77%). At the time of diagnosis, ages ranged from 1 day to 36 months (median 6.5 months). The diagnosis was delayed in one patient (PCG 44). Suspicion of primary congenital glaucoma was based on cornea enlargement (100%) and associated hazy corneas (80%), photophobia and blepharospasm (72%), and tearing (64%). Horizontal corneal diameters, measured under general anesthesia, ranged from 12 to 15 mm with an average of 13.25 ±0.9 mm. The mean preoperative intraocular pressure before the first surgical intervention was 25.4±7.1 mmHg. The average cup/disc size ratio was 0.36±1.2. Goniotomy was performed as the initial surgical procedure in 29 eyes (42%), combined trabeculotomy-trabeculectomy in 17 eyes (24.6%), trabeculectomy in seven eyes (10%), trabeculotomy in six eyes (8.7%), and surgical data were not available for eight eyes (11.6%). Forty-two eyes (60.8%) needed more than one surgery, and eight eyes (11.5%) required the implantation of the Ahmed valve.

### Analysis of *CYP1B1* mutations

**Figure 1:**
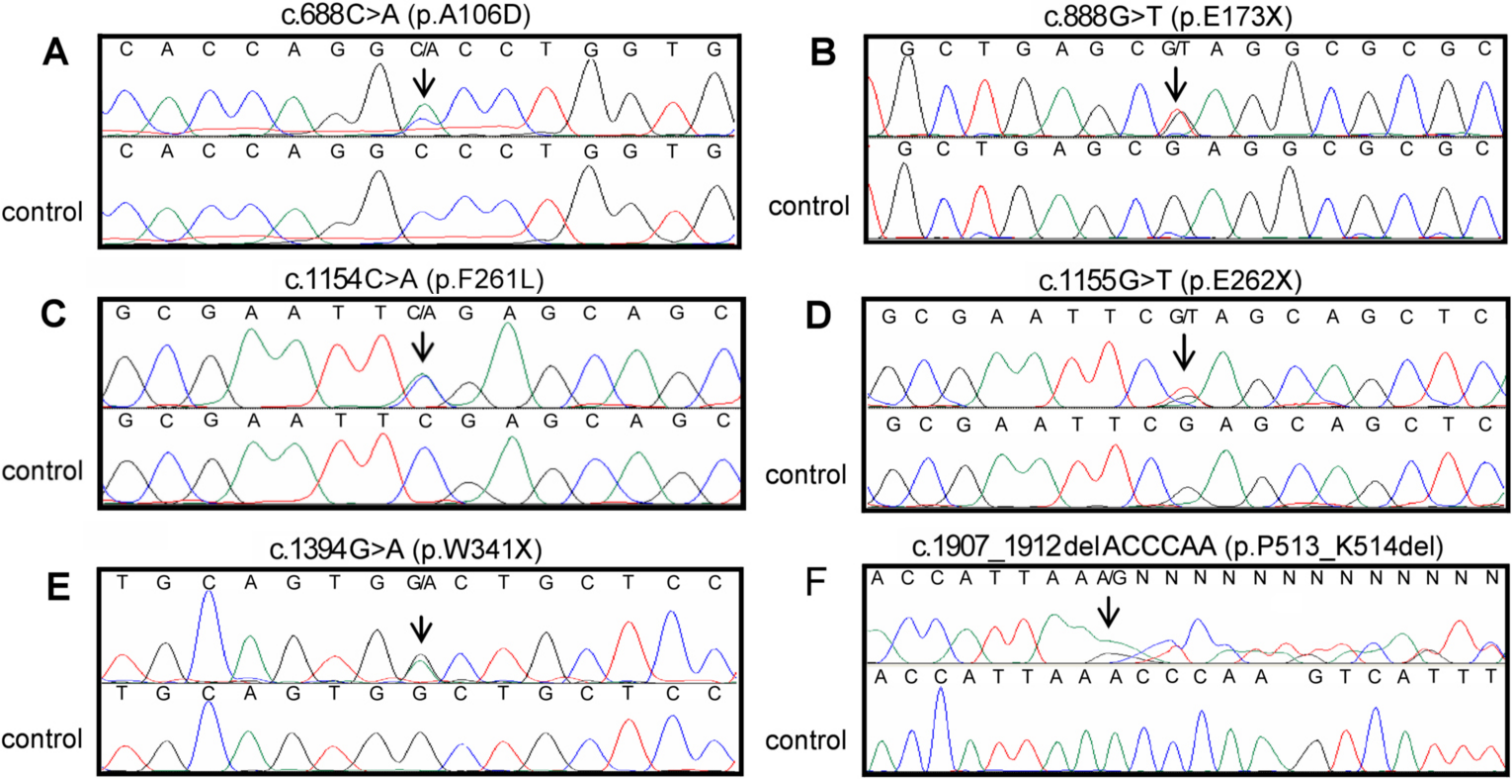
Detection of six novel *CYP1B1* mutations in Spanish PCG families by direct PCR DNA sequencing. Sequencing results of probands from families PCG 45 (**A**), PCG 28 (**B**), PCG 1 (**C**), PCG 29 (**D**), PCG 34 (**E**), and PCG 32 (**F**). Control electropherograms are shown for comparison purposes. The arrows indicate the position of mutations.

Mutations in the promoter region and the three exons of *CYP1B1* were analyzed by direct PCR sequencing in a total 109 people, which consisted of 39 affected by PCG and 70 unaffected relatives of the patients. No mutations segregating with the disease were found in the promoter region. We identified a total of 16 distinct DNA mutations in 13 (34.2%) of the 38 unrelated index cases: six transitions (2 G>A and 4 C>T), six transversions (3 C>A, 1 T>A and 2 G>T), three small deletions, and one small duplication (Table 2 and Figure 1). The transitions and transversions predicted nine missense (P52L, G61E, Y81N, A106D, F261L, R390S, P400S, P437L, and R469W) and three nonsense (E262X, W341X, and E173X) amino acid substitutions (Table 2 and Figure 1). On the other hand, the conceptual translation of one nucleotide long and 13 nucleotide long deletions (c.906delG and c.1435_1447delGAGTGCAGGCAGA, respectively) as well as one 10 nucleotide long duplication (c.1571_1580dupTCATGCCACC) resulted in three frameshift mutations followed by stop codons in the new reading frame (A179fsX18, T404fsX30, and R355fsX69, respectively; Table 2). Besides, the six nucleotide long deletion (c.907_912delACCCAA) predicted a two amino acid deletion, K513_P514del (Table 2 and Figure 1). To the best of our knowledge, 6 of the 14 mutations were novel (A106D, E173X, F261L, E262X, W341X, and K513_P514del; Figure 1). None of these six novel mutations was found in 325 (650 chromosomes) unrelated control subjects. The highest allelic frequency among PCG index cases with *CYP1B1* mutations corresponded to mutations T404fsX30 (23.0%) and R355fsX69 (11.5%; Table 2).

**Table 2:** *CYP1B1* mutations identified in Spanish PCG patients.

**Nucleotide change^a^**	**Amino acid change**	**Allelic frequency^b^ (%)**	**Exon**	**Background haplotype^c^**	**Reference**
c.526C>T	P52L	3.8	2	RAVN	[26]
c.553G>A	G61E	7.7	2	RAVN	[4]^d^
c.612T>A	Y81N	3.8	2	GSLN	[25]^e^
c.688C>A	A106D	3.8	2	RAVN	This work
c.888G>T	E173X	3.8	2	ND	This work
c.906delG	A179fsX18	3.8	2	RALS	This work
c.1154C>A	F261L	3.8	2	RALS	This work
c.1155G>T	E262X	3.8	2	RAVN	This work
c.1394G>A	W341X	3.8	2	RAVN	This work
c.1435_1447delGAGTGCAGGCAGA	R355fsX69	11.5	3	RAVN	[17]^f^
c.1539C>A	R390S	3.1	3	RAVN	[60]^g^
c.1569C>T	P400S	3.1	3	ND	[18]^h^
c.1571_1580dupTCATGCCACC	T404fsX30	23	3	RAVN	[61]^i^
c.1681C>T	P437L	3.8	3	RAVN	[13]^j^
c.1776C>T	R469W	7.7	3	RAVN	[12]^k^
c.1907_1912del ACCCAA	P513_K514del	7.7	3	RALN	This work

^a^Mutations were named based on cDNA reference sequence U03688 [39]. The first nucleotide of the transcription initiation site is denoted as nucleotide +1 according to Tang and coworkers [40]. ^b^The data from this column are the allelic frequency among PCG probands with *CYP1B1* mutations. ^c^The haplotypes are for the four common coding SNPs (R48G, A119S, V432L and N453S). These mutations have been described as follows: ^d^3987G>A; ^e^4046T>A; ^f^1410–1422delGAGTGCAGGCAGA; ^g^1514C>A; ^h^c.1198C>T; ^i^1546–1555dupTCATGCCACC; and ^j^1656C>T; ^k^1751C>T.

**Figure 2:**
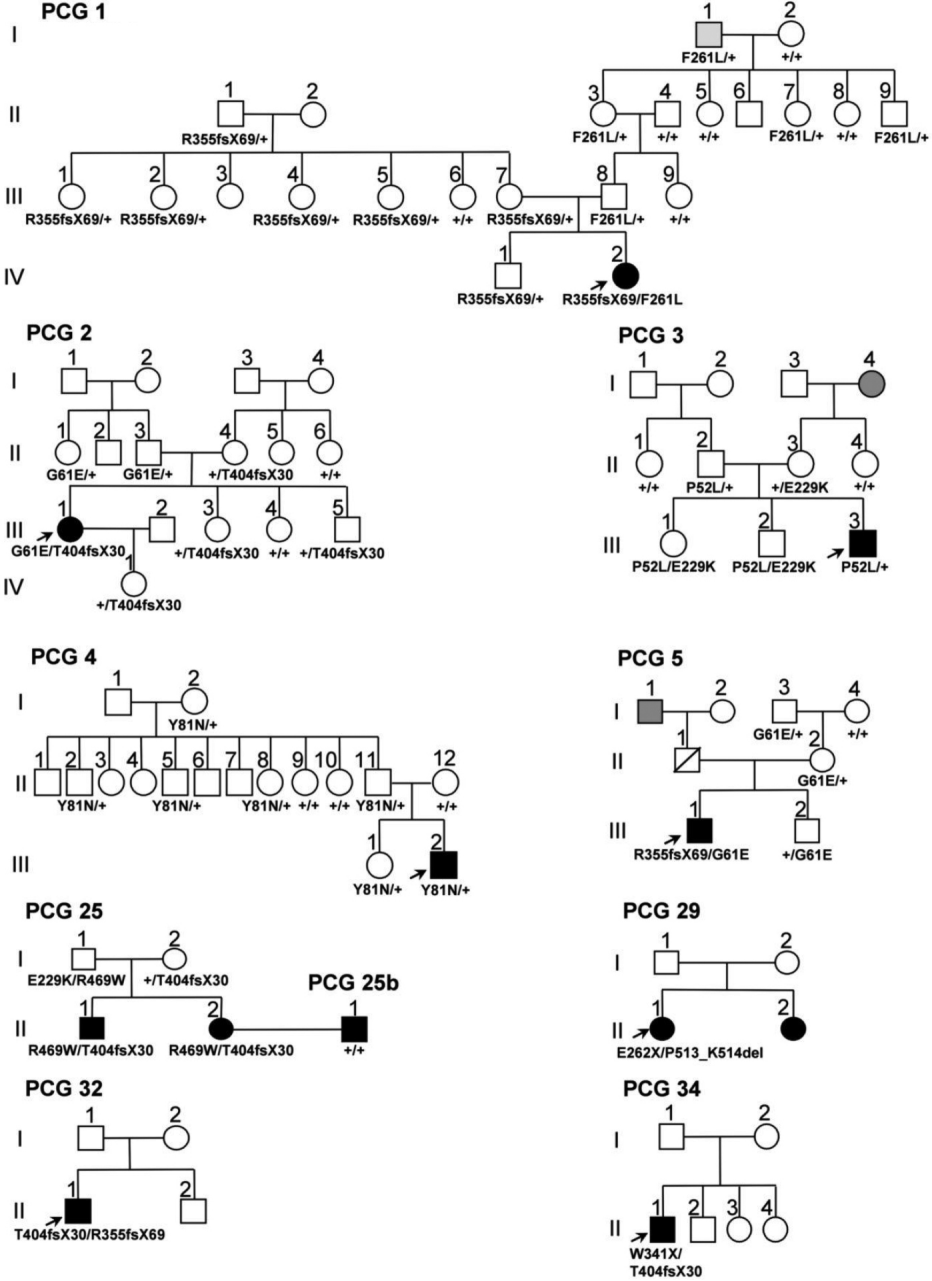
Pedigrees of PCG Spanish families with *CYP1B1* mutations. Genotypes are indicated below the symbols. DNA samples were not available from the parents of probands in families PCG 29, PCG 32, and PCG 34. Therefore, their genotypes could not be determined. Arrows show the probands. Black and gray symbols indicate PCG and OHT phenotypes, respectively. +: wild-type allele.

Most of these mutations were found in compound heterozygous subjects (85.7%). Nonetheless, we identified two index cases (14.3%) that were heterozygous for mutations P52L and Y81N (families PCG 3 and PCG 4, respectively; Figure 2). In contrast and as expected, relatives of these two patients who were heterozygotes for mutations Y81N and P52L were phenotypically normal (Figure 2). An intriguing finding was that three compound heterozygotes did not show any type of glaucoma at the time of the study. Two of them were siblings carrying mutations P52L and E229K (family PCG 3, subjects III:1 and III:2; Figure 2) while the third relative carried mutations E229K and R469W (family PCG 25, subject I:1; Figure 2). The age of these patients at the time of the study ranged from 28 to 58 years. Therefore, they likely represent cases of incomplete penetrance, which has been well documented in PCG [2,4,12,41]. It is interesting to note that mutation E229K was present in the three cases of incomplete penetrance detected in this study.

Mutations R355fsX69 and F261L were identified in the compound heterozygous state in the index case of family PCG 1 (IV:2) who manifested the disease at the time of birth (Figure 2). These mutations were also present in the heterozygous state in 11 unaffected carriers from the same pedigree. However, the oldest F261L carrier (I:1; 83 years old at the time of the study) was diagnosed with ocular hypertension (OHT) during the study, although we cannot rule out that this finding was merely a coincidence. Similarly, several normal heterozygous carriers of mutations G61E and T404fsX30 were identified in families PCG 2 and PCG 5.

Family PCG 25 also presented two affected siblings who who were compound heterozygotes with the same genotype (R469W/T404fsX30; Figure 2). As previously mentioned, their father could represent a case of incomplete penetrance. The affected woman, II:2, married an unrelated blind man diagnosed with PCG (PCG 25b, Figure 2) who had no mutations in either *CYP1B1* or *MYOC*. Two affected siblings were present in family PCG 29, the index case was a compound heterozygote (E262X/P513_K514del), but we were unable to perform DNA analysis in her affected sister (Figure 2). The index cases from families PCG 32 and PCG 34 were also compound heterozygotes. We ruled out the presence of mutations in the coding region of *MYOC* in all the families (data not shown).

### Haplotype analysis

Analysis of 4 coding common *CYP1B1 *SNPs (R48G, A199S, V432L, and N453S) revealed that RAVN was the most common haplotype (65.6%) among the Spanish patients who carried *CYP1B1 *mutations. In addition, RAVN was the haplotype background in 10 of the 16 identified mutations (Table 2).

### Evaluation of novel mutated positions by multiple sequence alignment

**Figure 3:**
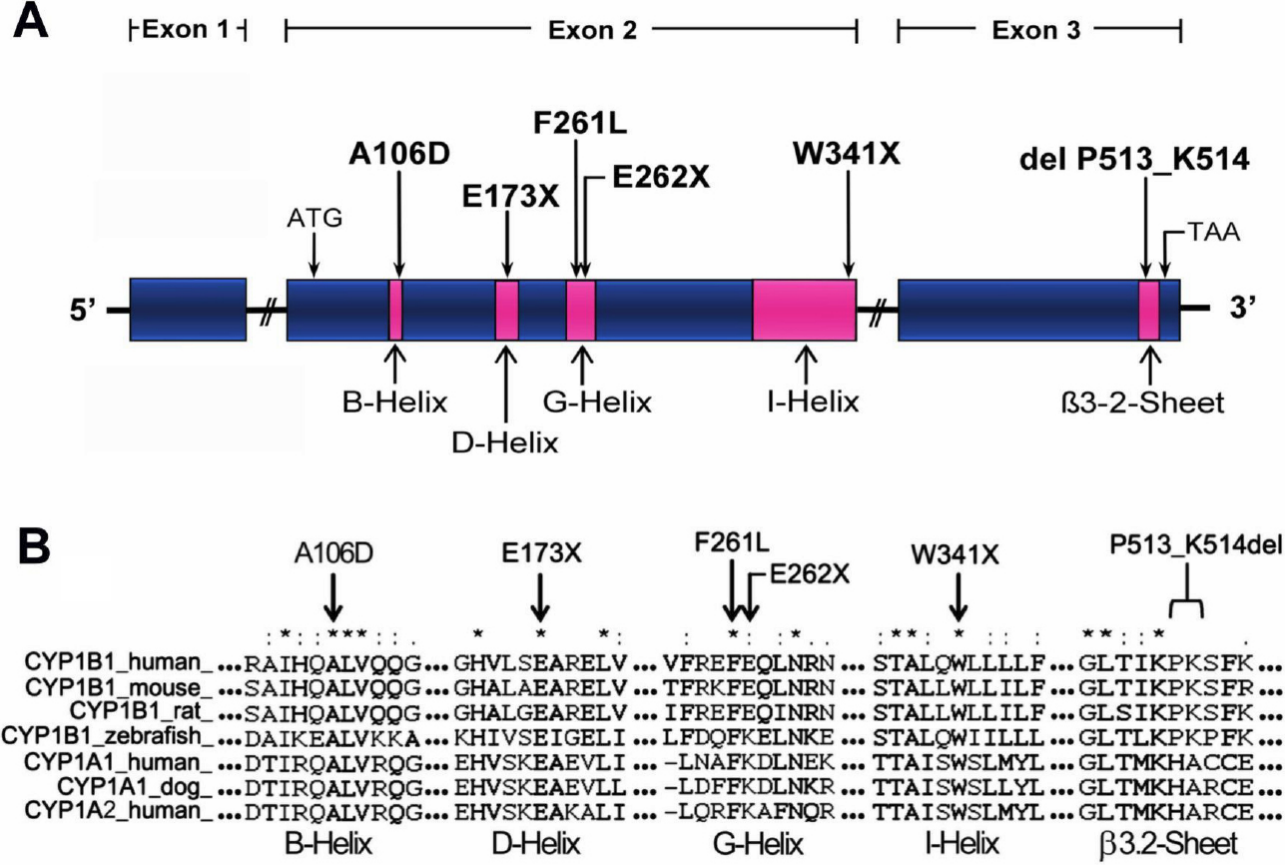
Location of six novel *CYP1B1 *mutations found in PCG patients. **A**: Scheme of the *CYP1B1 *gene showing the position of the novel mutations identified in this study. The three exons are represented by boxes, and different conserved structural domains encoded by them are depicted in pink. **B**: Multiple amino acid sequence alignment of CYP1B1, CYP1A1 and CYP1A2 from different species. The three exons are represented by boxes, and different conserved structural domains encoded by them are depicted by different patterns and shadings. Sequence alignment was generated by ClustalW. Arrows indicate the residues affected by mutations. Different structural domains of the cytochrome P450 superfamily are indicated. Asterisks indicate amino acid positions at which all query sequences are identical. Amino acid positions at which all the analyzed sequences had amino acids that were chemically similar are denoted by two dots (:). One dot denotes amino acid positions with weak chemical similarity (.).

To evaluate the degree of phylogenetic conservation of the novel mutated amino acid residues and the possible impact of the novel mutations in the structure and function of the protein, we compared the amino acid sequences of some mammalian and fish CYP1B1 enzymes and also two of the mammalian members of the well characterized CYP1A1 and CYP1A2 subfamilies (Figure 3). All the novel mutations affected highly conserved amino acid residues (Figure 3). The three nonsense mutations were predicted to truncate the protein at helices D, G, and I whereas the deletion of two amino acids (P513_K514del) affected the beta 3.2-sheet. These mutations are likely to produce a complete loss-of-function of the protein. The two novel missense mutations were located in helices B (A106D) and G (F261L; Figure 3).

### Functional analysis of CYP1B1 mutations

**Figure 4:**
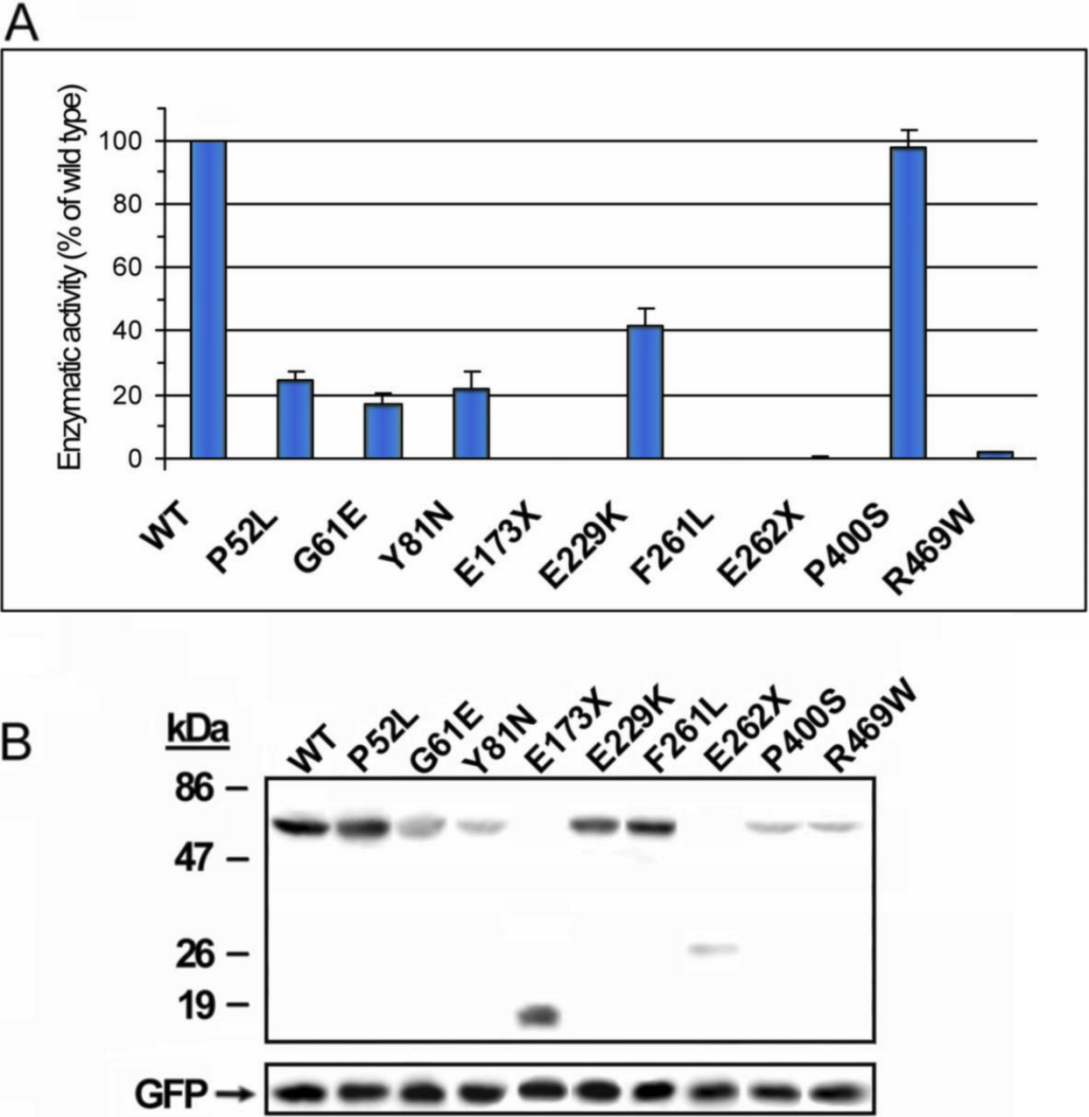
Effect of *CYP1B1* mutations found in Spanish PCG patients on CYP1B1 catalytic activity. **A**: cDNA constructs encoding the different mutations were transiently expressed in HEK-293T cells. Transfection efficiency was assessed by co-transfection with a cDNA construct encoding GFP. The EROD activity, expressed as a percentage of the activity of the wild-type protein, was measured as indicated in Methods. Error bars represent the SEM of triplicate experiments. **B**: Protein levels of the different CYP1B1 mutants present in transiently transfected HEK-293T cells 2 h after the enzymatic assay are shown as a control of protein expression. CYP1B1 polypeptides, tagged with the myc epitope at their COOH-terminal end, were detected by western immunoblot using a monoclonal anti-myc antibody (Santa Cruz). GFP was detected using an anti-GFP antibody (Santa Cruz).

To experimentally assess the effect of the mutations on the function of CYP1B1 and to analyze whether different levels of enzymatic activity correlate with the phenotype, we analyzed their enzymatic activity and protein stability. We were able to clone a total of nine mutations (three of them novel) by site-directed mutagenesis as described in the Methods. CYP1B1 enzymatic activity was estimated by determining the EROD activity in transfected HEK-293T cells with a fluorimetric assay at 2 h after addition of the substrate. Eight mutants showed reduced enzymatic activity compared with the wild-type protein while P400S showed normal activity (Figure 4A). Variants displaying an activity below 1% of the wild-type (E173X, F261L, E262X, and R469W) were classified as null alleles, and those with enzymatic activity ranging from 20% to 40% of wild-type CYP1B1 (P52L, G61E, Y81N, and E229K) were considered hypomorphic variants (Figure 4A). Detection of CYP1B1 in cells by western blot analysis 2 h after the enzymatic assay showed reduced protein levels for the following CYP1B1 mutants: G61E, Y81N, E262X, P400S, and R469W (Figure 4B). GFP analysis showed that there were no significant differences in transfection efficiency. These data indicate that although mutation P400S does not reduce the catalytic activity, it impairs protein stability, likely resulting in reduced enzymatic activity levels over time.

**Figure 5:**
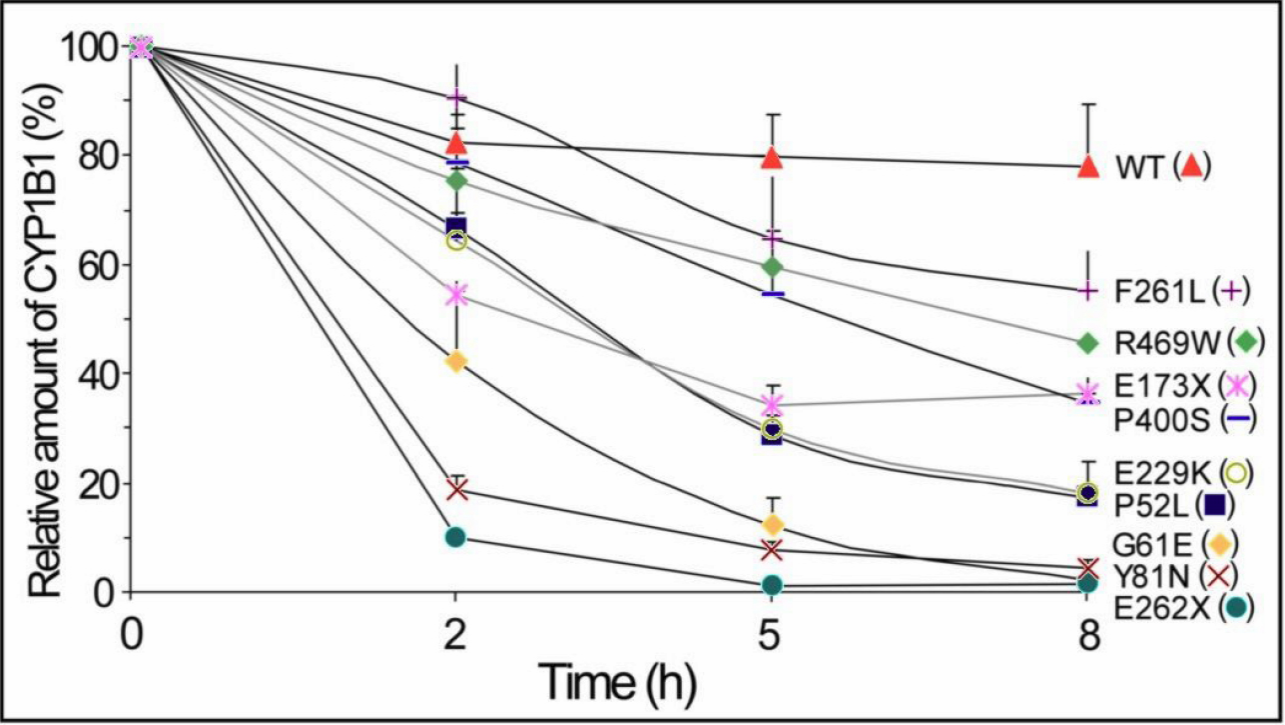
Time course stability of *CYP1B1* mutant polypeptides found in Spanish PCG patients. cDNA constructs encoding the different mutations were transiently expressed in HEK-293T cells. Cells were treated with cycloheximide, a protein synthesis inhibitor, and the amount of the different CYP1B1 polypeptides, tagged with the myc epitope at their COOH-terminal ends, were determined by densitometry of the signals detected in western blots using an anti-myc monoclonal antibody (Santa Cruz) at the indicated time points. Transfection efficiency was assessed by co-transfection with a cDNA construct encoding GFP. When required, CYP1B1 protein levels were corrected for transfection efficiency. Relative amounts of CYP1B1 are expressed as a percentage of levels at time 0 h. Error bars represent the SEM of triplicate experiments.

We then specifically investigated the protein stability of these mutants using transiently transfected HEK-293T cells that were treated with the protein synthesis inhibitor, cycloheximide, at various time points. Cells appeared viable upon microscopic examination at the latest time point used (8 h). We observed that the amount of all the CYP1B1 variants decreased upon exposure to cycloheximide, but the degradation of all the mutants was significantly more pronounced than that of the wild-type protein (Figure 5). Eight hours after cycloheximide exposure, the relative amount of wild-type CYP1B1 was 78% of the initial value (Time 0) while the protein level for three mutants was lower than 5% (G61E, Y81N, and E262X; Figure 5). A second group of mutants (P52L and E229K) showed protein levels close to 20% of the initial value. The amount of E173X, P400S, and R469W was around 40% while F261L showed the highest relative value among mutants (55%; Figure 5). Interestingly, although the last mutation did not strongly affect the stability of the polypeptide chain after 8 h of cycloheximide treatment, it completely inactivated the catalytic activity. However, mutation P400S, which did not impair the catalytic activity, significantly reduced the CYP1B1 protein levels to about 35% of the initial value. These data show that the CYP1B1 mutations associated with PCG impair the function of the cytochrome P450 molecule by affecting their catalytic activity and/or protein stability to different extents, thus supporting that different levels of residual CYP1B1 activity might underlie the development of the disease and phenotypic variability.

### Genotype-phenotype correlation

To analyze the genotype-phenotype relationship, we first compared the clinical features between PCG patients who carried *CYP1B1* mutations and those with no identified *CYP1B1* or *MYOC* mutations (Table 3). The percentage of bilateral cases, age at diagnosis, and male/female ratio did not differ significantly between the two groups. The mean IOP values in the affected eyes were higher among mutation carriers (26.8±7.8 mmHg) than among non-carriers (23.1±7.9 mmHg), although the differences were not significant (p=0.083). We then compared clinical parameters between patients with two *CYP1B1* null alleles and the rest of the carriers. Interestingly, the first group was of a younger mean age at diagnosis that those who carried at least one hypomorphic allele (0.6±0.9 months versus 17.3±15.2 months, respectively; p<0.05). Although patients with two null alleles showed higher mean IOP values at diagnosis and higher mean C/D ratios than patients with at least one hypomorphic allele, the differences were not significant. There were no significant differences noted for the three groups of patients with regard to the mean number of filtration surgeries required to control IOP (Table 3). All these data indicate that *CYP1B1* null mutations are associated with the severe PCG phenotypes mainly characterized by a very young age at diagnosis.

**Table 3:** Clinical features of patients with either *CYP1B1* mutations or no detected mutations.

**Family (Patient number)**	***CYP1B1* mutation**	**Gender/ laterality**	**Age at diagnosis (months)**	**^a^IOP (mm Hg) at diagnosis (OD/OS)**	**Initial surgery (OD/OS)**	**Number of glaucoma surgical interventions (OD/OS)**	**Age at last follow up (years)**	**Visual Acuity (Snellen chart) (OD/OS)**	**Corneal status (OD/OS)**	**Cup/disc ratio (OD/OS)**
PCG 2 (II:1)	p.G61E/p.T404fsX30	F/B	1	28/40	G/G	4/2	31	0.8/HM	C/C	ND/ND
PCG 21 (III:1)	p.R390S/p.A179fsX18s	M/B	2	ND/ND	G/G	>1/>1	30	A/1	A/C	A/0.4
PCG 5 (III:1)	p.R355fsX69/p.G61E	M/B	24	28/26	G/G	>1/>1	26	1/1	C/C	0.3/0.3
PCG 3 (III:3)	p.P52L/+	M/B	ND	ND/ND	G/G	2/2	15	0.3/LP	C/MSS	0.3/0.8
PCG 4 (III:2)	p.Y81N/+	M/U (OS)	23.5	16/30	-/CTT	0/1	6	0.9/1	C/C	0.2/0.2
PCG 1 (IV:2)	**p.R355fsX69/p.F261L**	F/U (OD)	0	23/12	-/CTT	1/0	3	1/1	C/C	0.2/0.2
PCG 25 (II:1)	**p.R469W/p.T404fsX30**	M/B	0	25/37	G/G	2/1	30	1/0.7	ND/ND	ND/ND
PCG 25 (II:2)	**p.R469W/p.T404fsX30**	F/B	0	ND/ND	ND/ND	ND/ND	32	LP/LP	ND/ND	ND/ND
PCG 27	**p.T404fsX30/p.R355fsX69**	M/B	2	27/29	T/T	2/2	4	0.25/0.15	C/MSS	0.8/UA
PCG 28	**p.E173X/p.P400S**	M/B	0	32/30	T/T	3^b^/3^b^	8	0.3/0.3	C/C	0.8/0.9
PCG 29	**p.E262X/p.P513_K514del**	F/B	2	18/24	T/T	4^b^/3^b^	6	0.9/A^c^	C/C	0.6/UA
PCG 32	**p.T404fsX30/p.P513_K514del**	M/B	1	38/39	G/G	4/2	29	ND/ND	CCE/C	ND/ND
PCG 34	**p.W341X/p.T404fsX30**	M/B	0	20/20	G/G	5/2	38	0.5/0.5	C/C	ND/ND
PCG 45	p.P437L/p.A106D	F/B	36	16/32	T/T	2^b^/2^b^	ND	0.4/0.6	ND/ND	ND/ND
PCG 26	-	F/B	0	35/35	ND/ND	2/2	ND	ND/ND	ND/ND	ND/ND
PCG 10	-	M/B	9	30/30	G/G	3/2	30	0.05/LP	CCE/CCE	UA/UA
PCG 22	-	F/U (OS)	18	ND/ND	-/Tt	0/1	30	1/LP	C/CCE	0.3/UA
PCG 11	-	F/U (OS)	ND	ND/ND	-/G	0/1	27	1/1	C/C	0.3/0.3
PCG 9	-	M/B	0.5	ND/ND	CTT/CTT	3/3	24	A^d^/A^e^	^d^/^e^	^d^/^e^
PCG 15	-	M/B	4	20/20	Tt/Tt	1/1	24	1/1	C/C	0.2/0.2
PCG 6	-	M/U (OS)	15	12/20	-/Tt	0/1	24	1/1	C/C	0.2/0.2
PCG 13	-	M/U (OS)	4	ND/ND	-/G	0/2	22	1/A^e^	C/^e^	0.3/^e^
PCG 8	-	M/B	2.5	22/22	CTT/CTT	1/1	17	1/1	C/C	0.2/0.2
PCG 14	-	M/B	1	22/22	CTT/CTT	1/1	16	1/0.7	C/MSS	0.3/0.4
PCG 7	-	M/B	5.5	20/20	CTT/CTT	2/1	12	0.3/LP	C/CCE	UA/UA
PCG 12	-	M/B	15	22/24	CTT/CTT	1/1	12	0.8/0.8	MSS/MSS	0.3/0.7
PCG 16	-	M/U (OD)	0.6	23/10	CTT/-	1/0	11	0.5/1	C/C	0.2/0.2
PCG 17	-	M/U (OS)	6	10/20	-/CTT	0/1	5	1/0.05	C/MSS	0.3/0.3
PCG 18	-	M/U (OD)	1	17/10	CTT/-	1/0	5	0.5/1	C/C	0.2/0.2
PCG 19	-	F/B	28.5	20/20	CTT/CTT	1/1	3	ND/ND	C/C	ND/ND
PCG 25b	-	M/B	ND	ND/ND	ND/ND	ND/ND	29	A/A	ND/ND	ND/ND
PCG 30	-	M/B	0	23/28	G/G	3/2	8	0.7/0.5	C/C	0.4/0.4
PCG 33	-	M/B	3	26/35	G/G	4/4	27	LP/LP	KB/PB	ND/ND
PCG 35	-	F/B	3	20/20	G/G	3/3	28	0.5/0.5	C/C	ND/ND
PCG 36	-	M/B	4	15/15	G/G	3/1	2	ND/ND	C/C	ND/ND
PCG 41	-	F/B	10	40/40	G/G	3/5	ND	ND/ND	ND/ND	0.9/0.9
PCG 42	-	M/B	4	38/30	G/T	3/2	ND	ND/ND	ND/ND	0.8/0.8
PCG 43	-	M/B	7	22/30	ND/ND	ND/ND	ND	ND/ND	ND/ND	0.2/0.3
PCG 44	-	M/B	48^f^	13/24	G/G	>1^b^/>1^b^	ND	0.5/0.4	^d^/CCE	UA/UA

Visual acuity was evaluated with a Snellen chart. Genotypes combining two *CYP1B1* null alleles are indicated in bold. A/HM/LP: amaurosis/hand movements/light perception; B/U: bilateral/unilateral; C/CCE/MSS: clear/chronic corneal edema/mild stromal scarring; CTT/G/T/Tt: combined trabeculotomy-trabeculectomy/goniotomy/trabeculectomy/trabeculotomy; KB/PB: keratopathy in band/peripheral defect; F/M: female/male; ND: not determined; OD/OS: right eye/left eye; UA: unassessable; ^a^IOP under general anesthesia; ^b^including Ahmed valve; ^c^persistent fetal vasculature unrelated to glaucoma; ^d^ptisis bulbi; ^e^eviscerated eye; ^f^delayed diagnosis.

## Discussion

### *CYP1B1* mutations in primary congenital glaucoma in Spain

Human *CYP1B1* has been implicated in primary congenital glaucoma in different human populations. However, the role of this gene in Spanish patients has not yet been investigated, and the number of studies performed in European populations is still low. Here, we report the first molecular and functional analysis of *CYP1B1* mutations in Spanish PCG patients. The findings, particularly those arising from functional studies, may have some general implications for understanding the pathogenesis of the disease. Our data show that around 34% of the studied index cases with autosomal recessive PCG carry mutations in this gene. This value is within the range found in groups of patients from different countries, around 10% in Mexico [42] and Ecuador [43]; 20% in Indonesia [17], Australia [18], and Japan [19]; around 40% in Turkish patients [6]; approximately 50% in Brazil [20] and France [21]; and about 100% in consanguineous Saudi Arabian [4] and Slovakian Gypsy [22] patients. These data illustrate that the contribution of defects in this gene varies significantly among human populations, which highlights that analyzing large groups of PCG from different ethnic backgrounds is required to ascertain the role of this gene in specific human groups. We also found an elevated proportion of compound *CYP1B1 *heterozygosity and allelic heterogeneity among Spanish patients. In this regard, a total of 16 different mutations were identified. To the best of our knowledge six of them were novel.

Mutation G61E has been previously identified in PCG patients from Saudi Arabia [2], Morocco [44], Mexico [42], and Turkey [6]. Variant Y81N was identified in Spanish [26] and French [25] POAG cases. Mutation P400S has previously been reported in the homozygous state in only one Australian PCG patient of Indian descent [18]. Interestingly, the father of this Australian PCG case was diagnosed with juvenile onset open-angle glaucoma and was heterozygous for this mutation, thus supporting the pathogenicity of this variant and showing that it is associated with different glaucoma phenotypes. Mutation P52L was initially described in a Spanish patient with POAG and also in a 66-year-old subject who was clinically normal at the time of the study [26]. Recently, it has been found as a loss-of-function mutation in German POAG patients and in one patient with hepatocellular adenoma [45]. Mutation P437L has been reported in PCG patients from Brazil [20], Turkey [12], and India [46]. It is noteworthy that these three PCG mutations (P400S, P52L, and P437L) were previously found in patients with POAG, showing their association with different glaucoma phenotypes.

### Haplotype background

Most of the mutations identified in this study were embedded in the same haplotype (RAVN). It has been reported that founder effects must have occurred for most *CYP1B1* mutations [47]. Our data agree with the founder effect hypothesis, but as this haplotype is the most common in the Spanish population, present in 37% of normal subjects (unpublished results), an extended haplotype analysis is required to confirm this hypothesis.

### Phylogenetic and functional analysis

All the novel mutations identified in this study affect highly evolutionary conserved amino acid residues and were predicted either to truncate the protein or to alter the physicochemical properties of the affected amino acids, supporting their pathogenic character. In that sense, mutation A106D changed a non-polar amino acid for a residue with a negative charge while F261L substituted a large and aromatic amino acid for an aliphatic residue. These mutations were located in helices B, D, G, and I and in the beta 3.2-sheet, which are structurally and functionally relevant. Helices B and D are relatively well conserved structurally [48]. The G helix forms part of the substrate access channel [49], and the I-helix of the heme-binding region contains catalytically important residues [49]. The COOH-terminal half of helix I and the beta 3.2-sheet belong to the most structurally conserved regions in cytochrome P450 [48]. Analysis of the EROD enzymatic activity demonstrated a complete loss-of-function of four mutants (E173X, F261L, E262X, and R469W), which clearly demonstrates that they are null alleles. In addition, the null alleles differed in protein stability. E262X was highly unstable while E173X, F261L, and R469W were moderately unstable. As all these mutations result in inactive enzyme variants, it is unlikely that the differences on stability may influence the phenotypic outcome. Most hypomorphic alleles (P52L, G61E, Y81N, and E229K) displayed reduced catalytic activity and protein instability (protein level after 8 h of protein synthesis inhibition less than 20% of the level at the initial time). Nonetheless, P400S showed normal catalytic activity and a significant reduction of protein stability. All these allele-dependent differences in enzymatic activity and protein stability could contribute to explain cases of incomplete penetrance as we will discuss later.

Few functional studies of pathogenic *CYP1B1* mutations have previously been reported. In accordance with our results, one of these studies found that mutants G61E and R469W have reduced enzymatic activity (50% and 30%, respectively) toward steroid hormones as substrates [33]. Regarding protein stability, Jansson and co-workers performed a study using membrane preparations obtained from bacteria expressing recombinant CYP1B1 mutants [33]. They found that G61E was much less stable than the wild-type protein (the CYP1B1 level decreased about 50%, after 24 h storage at 4 ºC). Unlike our results, however, they reported that R469W was as stable as the wild type enzyme. This discrepancy could be due to different experimental settings since in the present study we measured CYP1B1 levels in eukaryotic cells after inhibiting protein synthesis with cycloheximide. A second report also classified Y81N and E229K as hypomorphic alleles and proposed that these two sequence variants could function as risk alleles, which can lead to the development of glaucoma in the presence of modifier genes or environmental influence [34]. These two mutations have also been found to reduce protein stability, although while Y81N severely inhibits the turnover of various substrates, E229K impacts the ability to metabolize substrates moderately depending on the type of substrate [35].

### Incomplete penetrance and hypomorphic alleles

Incomplete penetrance has been associated with several *CYP1B1* mutations including G61E, R368H, D374N, R469W, g.4340delG, C209R, and g.4238del10 [2,4,41]. Interestingly, we observed three compound heterozygotes with incomplete penetrance in families PCG 3 and PCG 25 who carried at least one copy of the hypomorphic alleles P52L or E229K. In accordance with Bejjani and coworkers [4] and as mentioned before, we speculate that residual enzymatic activity from hypomorphic alleles in these subjects could be sufficient to maintain the normal phenotype either alone or in cooperation with compensating variants of unknown modifier genes and/or the increased *CYP1B1* expression induced by compounds present in the environment and/or the diet. In line with these ideas, it has also been proposed that reduced penetrance could be due to a dominant suppressor of the PCG phenotype, which is not linked genetically to *CYP1B1* [2], and/or to the inducibility of *CYP1B1* by environmental lipophilic agents to which individuals may have been exposed [33]. In fact, *CYP1B1* is inducible upon activation of the aryl hydrocarbon receptor by the binding of ligands such as polycyclic aromatic hydrocarbons [50,51] and by compounds found in certain vegetables [52]. According to the present results and to our hypothesis, mutation E229K accompanied by c.1064–1076del (likely a null allele) has also been identified in a normal carrier [47]. Incomplete penetrance, particularly although not exclusively associated with hypomorphic *CYP1B1* alleles, indicates that PCG is not a simple recessive trait and underscores its multifactorial character at least in a subset of patients. However, the glaucoma phenotype could be less variable and therefore, more predictable in patients who carry completely inactive *CYP1B1* alleles. Incomplete penetrance associated with partial loss of protein function originated by structural mutations has also been reported in diseases such as in familial retinoblastoma [53,54].

### Heterozygous *CYP1B1* mutations in primary congenital glaucoma

Another intriguing finding that apparently does not match a typical recessive pattern of inheritance is the presence of heterozygous *CYP1B1* mutations in PCG patients. This situation has been previously reported [35]. According to our data, mutation Y81N in the heterozygous state has also been described in a German PCG patient [47]. Moreover, E229K, R368H, and R469W were reported as the only mutant allele in Turkish PCG patients [6], and E229K has been identified in the heterozygous state in two unrelated French patients affected by PCG [21], in five Indian patients [55], in one Turkish case [6], and in one Caucasian patient [41]. Interestingly, some of these mutations in the heterozygous state are also associated with the milder POAG phenotype in patients from Spain [26], France [25], and India [24,56]. Recently, the presence of double heterozygous variants, *CYP1B1* and *FOXC1*, has been described in two PCG cases, although the role of possible digenism in disease causation is yet to be established [15]. Once again, defective variants of modifier genes and/or environmental compounds could cooperate with loss-of-function *CYP1B1* alleles to produce the disease phenotype. However, further work is required to unravel this point.

### Genotype-phenotype correlation

Previous studies have reported that the age at diagnosis in PCG patients with *CYP1B1* mutations is younger than for patients without mutations [57]. Our data show that although the disease was diagnosed earlier in carriers of *CYP1B1* mutations than in non-carriers, the differences found were not significant. We reasoned that patients with two null *CYP1B1* alleles, which means there is no CYP1B1 catalytic activity, could present more severe phenotypes than carriers of at least one hypomorphic allele, who likely conserve residual enzymatic activity levels. According to this hypothesis, our results show that PCG patients with a complete loss of CYP1B1 enzymatic activity display the most severe disease phenotype featured by very early mean onset of the disease (0.6±0.9 months). We also observed higher mean IOP values and optic disc excavations in this group of patients, but the differences were not statistically significant. In accordance with the idea of associating the worst phenotypes with null *CYP1B1* mutations, the percentage of severe phenotypes in at least one eye have been reported to be associated with various mutations ranging from 100% for a frameshift mutation (376insA) producing a truncated protein at codon 223 (null allele) to approximately 62%–83% for different missense mutations such as G61E, P193L, E229K, R368H, and R390C [55]. According to our data, two of these mutations (G61E and E229K) are hypomorphic alleles. Moreover, E229K has been found as the only *CYP1B1* allele in a PCG case with moderate goniodysgenesis [41]. Further studies with a larger number of patients will be required to confirm whether in fact the complete absence of CYP1B1 activity correlates with the worst phenotype.

The identification of a panel of 16 different *CYP1B1* mutations for the first time in Spanish PCG patients with allele dependent functional heterogeneity could provide a basis to better understanding of the disease as well as to improve genetic testing and genetic PCG counseling. This in turn will contribute to early diagnosis and prevention of blindness, which can result from a late diagnosis of PCG. Hopefully, this will reduce the burden of the afflicted families and will improve their quality of life since it has been shown that early and prompt surgical interventions in PCG patients leads to a better prognosis [58,59]. Finally, our data clearly indicate that other genetic and/or environmental factors remain to be identified, particularly in patients carrying hypomorphic mutations.
